# Biochemical characterization of the minimal domains of an iterative eukaryotic polyketide synthase

**DOI:** 10.1111/febs.14675

**Published:** 2018-10-25

**Authors:** Martin Sabatini, Santiago Comba, Silvia Altabe, Alejandro I. Recio‐Balsells, Guillermo R. Labadie, Eriko Takano, Hugo Gramajo, Ana Arabolaza

**Affiliations:** ^1^ Facultad de Ciencias Bioquímicas y Farmacéuticas Instituto de Biología Molecular y Celular de Rosario (IBR‐CONICET) Universidad Nacional de Rosario Argentina; ^2^ Facultad de Ciencias Bioquímicas y Farmacéuticas Instituto de Química de Rosario (IQUIR‐CONICET) Universidad Nacional de Rosario Argentina; ^3^ Manchester Centre of Fine and Specialty Chemicals (SYNBIOCHEM) Manchester Institute of Biotechnology (MIB) University of Manchester UK

**Keywords:** domain deconstruction, iterative PKS, PKS biochemistry, substrate specificity

## Abstract

Iterative type I polyketide synthases (PKS) are megaenzymes essential to the biosynthesis of an enormously diverse array of bioactive natural products. Each PKS contains minimally three functional domains, β‐ketosynthase (KS), acyltransferase (AT), and acyl carrier protein (ACP), and a subset of reducing domains such as ketoreductase (KR), dehydratase (DH), and enoylreductase (ER). The substrate selection, condensation reactions, and β‐keto processing of the polyketide growing chain are highly controlled in a programmed manner. However, the structural features and mechanistic rules that orchestrate the iterative cycles, processing domains functionality, and chain termination in this kind of megaenzymes are often poorly understood. Here, we present a biochemical and functional characterization of the KS and the AT domains of a PKS from the mallard duck *Anas platyrhynchos* (ApPKS). ApPKS belongs to an animal PKS family phylogenetically more related to bacterial PKS than to metazoan fatty acid synthases. Through the dissection of the ApPKS enzyme into mono‐ to didomain fragments and its reconstitution *in vitro,* we determined its substrate specificity toward different starters and extender units. ApPKS AT domain can effectively transfer acetyl‐CoA and malonyl‐CoA to the ApPKS ACP stand‐alone domain. Furthermore, the KS and KR domains, in the presence of *Escherichia coli* ACP, acetyl‐CoA, and malonyl‐CoA, showed the ability to catalyze the chain elongation and the β‐keto reduction steps necessary to yield a 3‐hydroxybutyryl‐ACP derivate. These results provide new insights into the catalytic efficiency and specificity of this uncharacterized family of PKSs.

**Abbreviations**
ACP: acyl carrier protein
AT: acyltransferase
DH: dehydratase
ER: enoylreductase
HR: high reducing
KR: ketoreductase
KS: β‐ketosynthase
NR: nonreducing
PKS: polyketide synthases

## Introduction

Polyketides are an important family of natural compounds which comprise a broad range of biological and pharmacological activities, including antibiotic, immunosuppressant, antitumor, antifungal, and antiparasitic agents. Overall, this covers a large variety of chemical entities such as polyethers, polyenes, polyphenols, macrolides, enediynes, and complex lipids 1, 2, 3.

The structural and functional diversity of these natural products is accomplished by exquisite chemical processes executed by megaenzymes called polyketide synthases (PKSs). PKSs are large multifunctional enzymes that exhibit diverse structural organization and have been classified as types I, II, and III. Type I PKSs contain, within a multifunctional polypeptide, all the enzymatic activities necessary for one cycle of β‐keto chain elongation and processing, and can be either modular (mostly in bacteria) or iterative (mostly in fungi). Iterative PKSs repeatedly reuse one set of enzymatic domains, whereas modular PKSs use enzymatic domains once in a serial and consecutive manner and are organized as large linear arrangements of modules 4.

The crucial aspect of polyketide molecule assembly is the formation of the carbon–carbon bond achieved by a decarboxylative Claisen condensation. This condensation reaction takes place in the active site of the ketoacyl synthase (KS) domain, where the starter unit or the growing polyketide chain is anchored via a thioester linkage (electrophile). Once the acyltransferase (AT) domain has transferred an α‐carboxyacyl‐CoA extender unit (usually malonyl‐ or methylmalonyl‐CoA) to the acyl carrier protein (ACP) domain (nucleophile), the KS catalyzes the condensation reaction between the electrophile and nucleophile to form a β‐ketoacyl‐ACP intermediate. Thus, all central stages in the carbon–carbon bond formation—the invariant part in every assembly step—are facilitated by only two enzymatic functions, KS and AT 5. Then, the growing carbon chain could be modified by three sequential reactions, where the ketoreductase (KR), dehydratase (DH), and enoylreductase (ER) activities optionally process the resulting β‐keto group of the condensation product. The variability in the degree of reduction in the keto group thus contributes to the chemical versatility of natural polyketide synthesis. Overall, this biosynthetic scheme shares many similarities with fatty acid synthesis, including the utilization of common precursors, similar chemistry, structure, and overall architectural design 4.

Until now, most efforts were focused on characterizing the modular type of bacterial PKSs, the 6‐deoxyerythronolide B synthase (DEBS), one of the best‐studied megaenzyme, and representing a prototypical assembly line 6, 7. Furthermore, the deep understanding of their biochemistry and structure has led to considering these enzymes as remarkable biosynthetic machines with a potential for structure‐based engineering of custom products. Several reports have proven the feasibility of the concept 8, 9, 10, 11, 12, 13, 14, 15, 16, and PKS engineering has emerged as a powerful tool to modify the activity of domains and the substrate specificity in order to generate structural diversity of the final product. Briefly, such modifications include replacing domains with those having higher substrate tolerance, introduction of key mutations to change substrate specificity, deleting or inserting domains, or deleting or inserting entire modules 10, 12. This concept and its implementation have been applied for the engineering of iterative fungal PKS 13 and it has been used for the engineering of an iterative megaenzyme such as the fungal FAS 14, 15, 16.

An alternative, although somewhat more restricted approach toward harnessing PKS for custom product synthesis, would be built on the basic idea of continuing searching, cataloging, and characterizing novel and less complex PKSs. With this rationale, we focused on iterative PKSs since they are structurally simpler than multimodular PKSs, facilitating their cloning, further genetic manipulation, heterologous expression, and protein purification.

Iterative PKSs use an unknown set of programming rules and the order of catalytic events are difficult to be deciphered from just examining their primary amino acid sequences 17, 18. In this study, we focused on a recently identified family of animal PKSs, for which no biochemical or enzymatic data had yet been reported. So far, the substrate specificity in terms of starters and extender unit election, or their catalytic efficiency has not been addressed to our knowledge. Thus, since the structure of the final product mainly depends on the KS and AT activities, and given that the AT, KS, and ACP are the minimal domains required for polyketide synthesis, hence called minimal PKS, we explored their functionality via domain deconstruction of the unique PKS annotated from *Anas platyrhynchos,* here named ApPKS. In this work, individual domains of ApPKS were reassembled *in vitro* and its substrate specificity was determined toward different starters and extender units, enabling the study of the synthetic contribution of each domain to the overall product formation.

## Results

### *In silico* studies and phylogenetic analysis of metazoan PKSs

**Figure 1:**
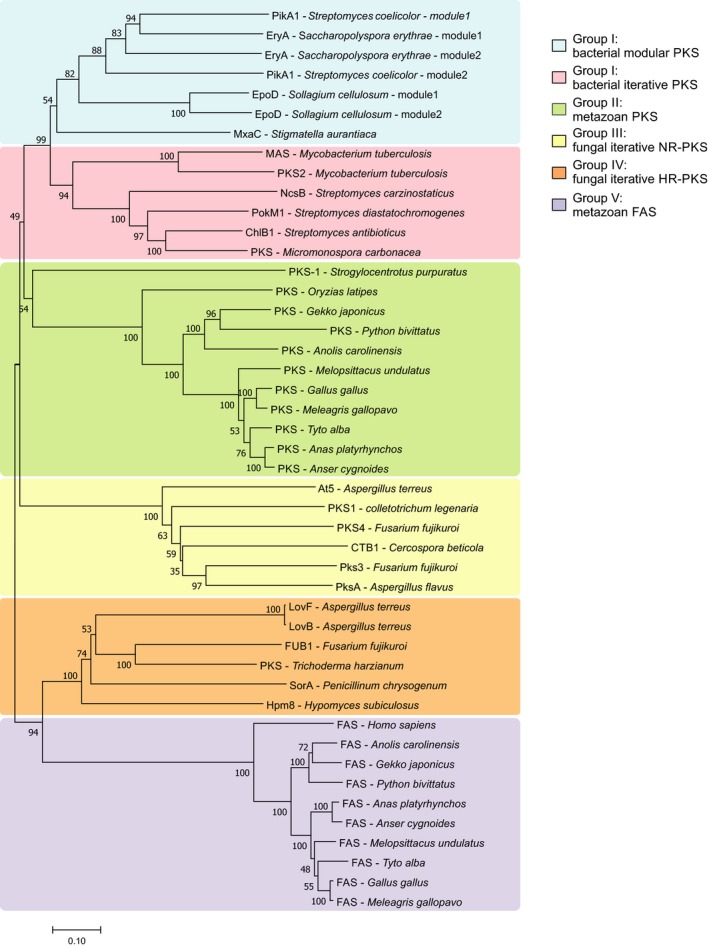
Phylogeny of polyketide synthases. Maximum likelihood tree based on KS‐AT didomain from representative type I PKS families including iterative and modular bacterial PKSs, NR, and highly reducing fungal PKSs, metazoan PKSs (birds and reptiles), and fatty acids syntases (birds, reptiles and human). Bootstrap values (based on 1000 replicates) are indicated at the tree nodes. The scale bar below denotes substitutions per site.

Animal PKSs were rarely explored, except for four recent examples: the echinoderm pks‐1 and pks‐2 isolated from *Strongylocentrotus purpuratus* 19, OlPKS from medaka fish *Oryzias latipes* 20, a modular polyketide synthase named PKS‐1 of *Caenorhabditis elegans* 21 and MuPKS from the budgerigar *Melopsittacus undulatus* 22. The recent characterization of the MuPKS products suggests that this enzyme functions by an iterative mechanism, which is part of a larger closely related group among of metazoan PKSs 22. In order to determine the phylogenetic relationship between metazoan PKSs with other PKSs, from species as distant as the fungi and bacteria, and with metazoan FAS, we selected a set of fungal and bacterial PKSs and several animal FAS to construct a phylogenetic tree based on a multiple sequence alignment of the KS‐AT didomain. Briefly, the amino acid sequences of 46 polypeptides were analyzed using mega (version 7) 23, refined by visual inspection, and finally, a phylogenetic tree was inferred by the maximum likelihood method. In this analysis, we mainly included well‐characterized iterative PKSs from fungi and bacteria; and we selected, among animal PKSs homologs, the predicted protein sequences from some members of the phylum Chordata, the previously mentioned Pks‐1 from *S. purpuratus* and the PKS from *O*. *latipes*. The results illustrated in Fig. 1 showed five clearly defined groups (I–V; Fig. 1). Group I includes bacterial PKSs, further subdivided into modular and iterative; group II comprised animal PKSs; group III is formed by nonreducing (NR) fungal iterative PKSs; group IV is represented by iterative high reducing (HR) fungal PKSs; and group V contains metazoan fatty acid synthases (FAS). This clear subdivision of the diversity of PKS enzymes indicates that the animal PKSs are monophyletic and phylogenetically distinct from animal FASs, being phylogenetically more closely related to bacterial PKSs than to metazoan fatty acid synthases. This observation is intriguing given the evolutionary distance between these two domains (Bacteria and Eukarya) and raises interesting questions regarding the evolutionary origin of animal *pks* genes.

On the other hand, predictive structural analysis showed that the selected metazoan PKSs (proteins of group II, Fig. 1) share a common domain arrangement, with the six consecutive domains: KS, AT, DH, ER, KR, and ACP. Similar to some bacterial PKS, for example, the mycoserosic acid synthase, Mas, from *Mycobacterium tuberculosis*, these proteins lack an integrated product release domain, such as a thioesterase domain, suggesting that a trans‐acting partner should be involved in the final product processing step. In the case of Mas, the AT PapA5 is required for transferring the tetramethyl‐branched fatty acid product attached to the Mas ACP to a phthiocerol acceptor molecule 24. The group III megaenzymes showed the conserved amino acid motives that define each of the proposed domain active sites. Furthermore, no C‐methyl transferase domain was detected; the ER domain was suggested to be inactive, in some of its members, based on the lack of the canonical NADPH‐binding motif 22.

Particularly, a single gene copy and synteny with neighboring genes was observed in avian homologs. Although, in some species, such as *Gallus gallus* and *Coturnix japonicum* in which synteny is maintained, there exist two *pks* gene copies coding for predicted protein sequences with 98% identity. Overall, the existence of mainly a single coding gene and a detailed inspection of the primary sequence of the annotated birds PKS proteins suggest that they may perform an iterative mechanism. For example, these enzymes lack the typical amino acid stretches that act as intermodule linkers. In modular PKS, interpolypeptide linkers consist of 80–130 amino acids at the C‐terminal of one module that interacts with a cognate 30–50 aminoacid sequence at the N terminus of the downstream module 8. The proposed iterative mechanism would be in line with the recently characterized MuPKS*,* whose product was identified as a highly unsaturated C14, C16, and C18 fatty‐acyl precursor of the yellow psittacofulvin pigment found in budgerigar feathers 22. MuPKS is the only example of an animal iterative PKS where the chemical structure of the product was established. However, no mechanistic or biochemistry studies were carried out with this or any other animal PKS megaenzymes.

Based on these observations, we chose as a model of study the unique PKS found in mallard duck (*A. platyrhynchos)*, hereafter named ApPKS, for further biochemical analysis via domain deconstruction.

### Design, expression, and purification of individual ApPKS domains

**Figure 2:**
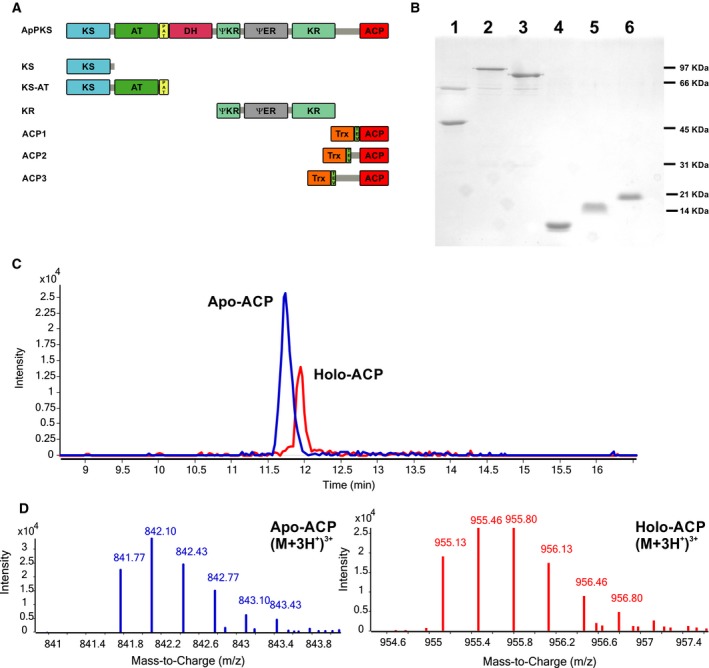
ApPKS deconstruction. (A) Schematic representation of ApPKS domain organization and all the constructions used in this study. (B) Coomasie‐stained SDS/PAGE of purified constructs after Ni^2+^ affinity purification. Lane 1, KS (47.1 KDa), lane 2, KS‐AT (97.6 KDa), lane 3, KR (87.8 KDa), lane 4, ACP1 (7.8 KDa), lane 5, ACP2 (13.6 KDa), and lane 6, ACP3 (20.9 KDa). All constructs were expressed as N‐terminal 6xHis‐tag fusion proteins with the exception of KS, which was expressed as C‐terminal his‐tag fusion. The ACP constructs were fusions to 6xHis‐thiorredoxin (trx; Table S1). TEV represent the TEV protease cleavage site and PAT stands for post‐AT Linker. In lanes 1, 2, and 3, the protein at 60 KDa corresponds to the chaperon GroEL obtained during the protein purification as by‐product. (C) LC‐MS chromatogram of the peptides from Apo and Holo forms of ACP1 after digestion with Trypsin and GluC. (D) Mass spectra of the Apo and Holo forms of ACP1 peptides, the ions shown correspond to the most abundant ion formed, (M + 3H^+^)^3+^.

In order to carry out a biochemical characterization of the minimal PKS activities (KS, AT, and ACP domains) from ApPKS, we first analyzed the primary sequence of the protein *in silico*. As previously mentioned*,* ApPKS (as well as the other members of group III PKS, Fig. 1) presents the complete set of KS, AT, DH, ψKR, ER, KR, and ACP domains (Fig. 2). Thus, the dissection of this protein into mono‐ or didomain fragments was based on a careful primary protein sequence examination, where the cut sites for protein deconstruction were guided by a variety of bioinformatics analyses including multiple sequence alignment, secondary structure prediction, and domain boundary prediction using the SBSPKS tool and the NCBI conserved domain service. The DNA sequences encoding for the selected recombinant domains used in this work (schematized in Fig. 2) were expressed in *Escherichia coli* BL21(DE3) or BAP1 (for ACP domain) strains, and the corresponding proteins were purified by affinity chromatography as either N‐ or C‐terminal 6xHis‐tag fusions (Fig. 2; Table S2).

Monodomain fragments included the KS and three versions of recombinant ACP (each differing at the N termini). ACP1 contains the sequence recognized as ACP with the SBSPKS tool, which completely excludes the ACP‐KR linker, this sequence corresponds to the core of the phosphopantetheine‐binding site, and its predicted structure is comparable to previously reported ACPs 25, 26; ACP2 spans through part of the ‘linker region’ containing a helix that was reported to be part of the ACP from module 2 of DEBS 25; and ACP3 contains the entire predicted linker region between the KR and ACP. The three different ACPs were expressed as insoluble proteins. Therefore, to circumvent this problem, each single ACP domain was finally cloned as N‐terminal 6xHis‐thioredoxin (HIS‐TRX) fusion protein. This HIS‐TRX tag was removed during protein purification by TEV protease‐mediated cleavage (see Experimental procedure). The Apo‐ACP/Holo‐ACP ratio for the three ACP versions mentioned above was approximately 0.4, and it was determined by calculating the area under the peaks of the LC‐MS/MS chromatogram as shown for ACP1 in Fig. 2C,D. Didomain fragments encompassed: (a) the KS‐AT, including the post‐AT linker, which was shown to be essential for the overall didomain activity 27; and (b) a recombinant KR segment that comprised the predicted ψKR, located upstream of the ER domain, which was reported to be a structurally important part of the KR domain 28. The KS domain, KS‐AT, and ψKR–ER–KR didomains were expressed as partially soluble proteins, therefore, the expression of these proteins was further assisted with a set of chaperons (GroEL, GroES, and Tig 29, 30) that improved their solubility, allowing higher purification yields (Table S2).

### Acylation and Transacylation reactions of Recombinant KS‐AT didomain

**Figure 3:**
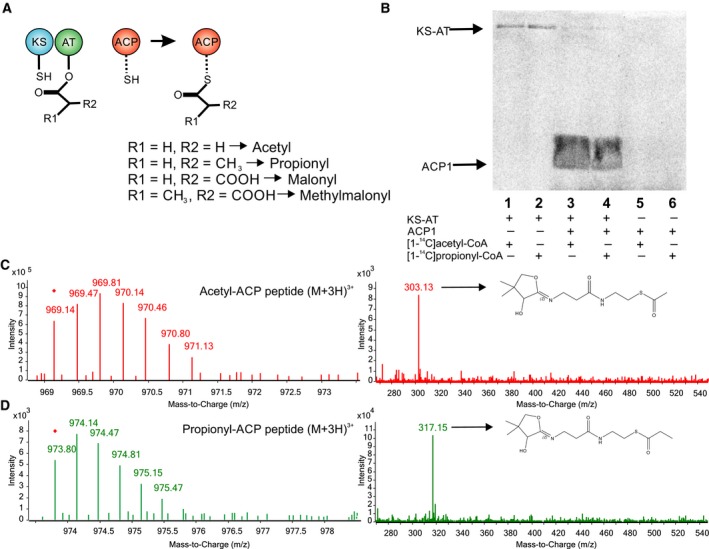
Acylation and transacylation of the KS‐AT didomain with different starter units. (A) Schematic representation of the transfer reaction of starter or extender units to ACP1 by KS‐AT. The starter units analyzed were acetyl‐CoA and propionyl‐CoA, and the extender units were malonyl‐CoA and methylmalonyl‐CoA. (B) SDS/PAGE autoradiography of purified KS‐AT and ACP1 coincubated with radiolabeled acetyl‐CoA or propionyl‐CoA. (C) Mass spectrometry analysis of the ACP formed in transacylations assay with acetyl‐CoA. Left panel, mass spectra of the ACP peptide bound to an acetyl group. The most abundant specie found was (M + 3H^+^)^3+^. Right panel, pantetheinyl ejection fragments observed during tandem mass spectrometry of the ion at *m/z* 969.14, the most abundant ion is at *m/z* 303.13, the pantetheinyl elimination of acetyl‐ACP. (D) Mass spectrometry analysis of the ACP formed in transacylations assay with propionyl‐CoA. Left panel, mass spectra of the ACP peptide bound to a propionyl group. The most abundant specie found was (M + 3H^+^)^3+^. Right panel, pantetheinyl ejection fragments observed during tandem mass spectrometry of the ion at *m/z* 973.80, the most abundant ion is at *m/z* 317.15, the pantetheinyl elimination of propionyl‐ACP.

The purified KS‐AT didomain and the stand‐alone ACP1 domain were initially assayed for characterizing the AT catalytic properties toward different starter and extender units (Fig. 3A). Thus, in order to determine the functionality of the AT domain in the presence of possible starter units acetyl‐CoA and propionyl‐CoA, we performed acylation and transacylation assays. For this, recombinant KS‐AT protein was incubated with [1‐^14^C]acetyl‐CoA or [1‐^14^C]propionyl‐CoA, with or without added ACP1. Figure 3B shows a radio‐SDS/PAGE where the KS‐AT didomain was readily acylated by both radioactive substrates in the absence of ACP1 (Fig. 3B, lane 1 and 2), suggesting that either the active site serine of the AT domain or the active site cysteine of the KS domain are covalently bound with the starter unit. However, in the presence of ACP1, and under identical assay conditions, the AT domain catalyzed the transfer of the acyl group toward the carrier domain (Fig. 3B, lane 3 and 4). Furthermore, no ACP1 labeling could be observed in the absence of KS‐AT didomain (Fig. 3B, lane 5 and 6).

**Figure 4:**
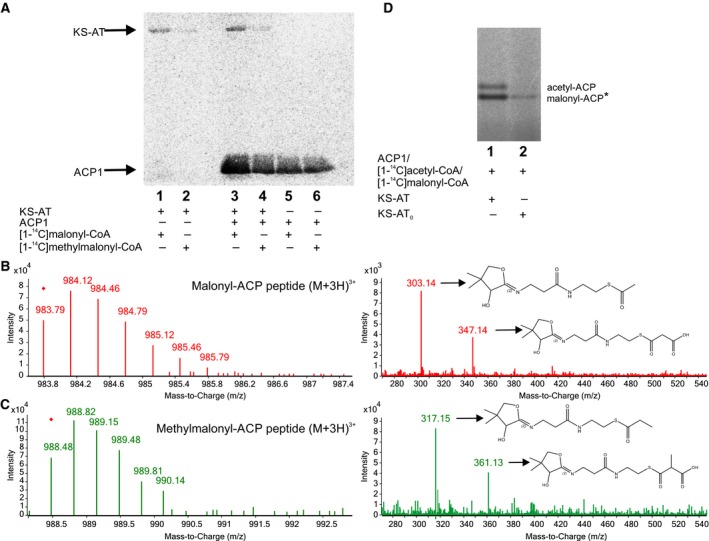
Acylation and transacylation of KS‐AT didomain with different extender units. (A) SDS/PAGE autoradiography of KS‐AT and ACP1 coincubated with radiolabeled malonyl‐CoA and methylmalonyl‐CoA. (B) Mass spectrometry analysis of the ACP formed in transacylation assays with malonyl‐CoA. Left panel, mass spectra of the ACP peptide bound to an acetyl group. The most abundant species found was (M + 3H^+^)^3+^. Right panel, pantetheinyl ejection fragments observed during tandem mass spectrometry of the ion at *m/z* 983.79, the ion at *m/z* 347.14 belongs to the pantetheinyl elimination of malonyl‐ACP. The high signal for ion at *m/z* 303.14 belongs to pantetheinyl elimination of acetyl‐ACP formed by decarboxylation induced by the collisional energy applied for MS/MS. (C) Mass spectrometry analysis of the ACP formed in transacylations assay with methylmalonyl‐CoA. Left panel, mass spectra of the ACP peptide bound to an acetyl group. The most abundant species found was (M + 3H^+^)^3+^. Right panel, pantetheinyl ejection fragments observed during tandem mass spectrometry of the ion at *m/z* 988.48, the ion at *m/z* 361.13 is the pantetheinyl elimination of methylmalonyl‐ACP. The high signal for ion at *m/z* 317.15 belongs to pantetheinyl elimination of propionyl‐ACP formed by decarboxylation induced by the collisional energy applied for MS/MS. (D) Conformational sensitive gel electrophoresis of the transacylation assay performed with KS‐AT and KS‐AT_0_. (*) lane 2: self‐malonylation of ACP1.

A similar experiment performed in the presence of the possible extender units [1‐^14^C]malonyl‐CoA and[1‐^14^C]methylmalonyl‐CoA showed that the KS‐AT didomain became labeled when coincubated with [1‐^14^C]malonyl‐CoA, and to a lesser extent with [1‐^14^C]methylmalonyl‐CoA (Fig. 4A, lane 1 and 2). The addition of ACP1 to each reaction mix led to the detection of the corresponding ACP1‐labeled species with both substrates (Fig. 4A, lane 3 and 4). However, a self‐acylation activity of the discrete ACP1 domain was observed (Fig. 4A, lines 5 and 6). Despite this phenomenon, the increase in labeling of ACP1 in the presence of KS‐AT (Fig. 4A, lane 3 versus line 5) suggested an additional AT‐mediated transfer reaction, which was more evident for the malonyl group. As reported previously, ACPs from type II FAS and PKS can be self‐loaded with carboxyacyl‐CoAs 31, 32. The ACP1 from ApPKS expressed as a stand‐alone protein exhibits this property as well. To our knowledge, this observation has not been reported before for type I PKS or FAS.

All acyl‐ACP1 species generated were confirmed by LC‐MS/MS (Figs 3C,D and 4B,C) using an adaptation of the method described by Dorrestein *et al*. 33. This method allows the identification of the ACP peptide containing the 4‐phosphopantetheine arm attached to an acyl group. The ions produced by the peptides are identified and selected for fragmentation by Collision‐Induced Dissociation (CID). The different pantetheinyl fragments attached to an acyl group that are released allow the identification of the acyl‐ACP species present in the samples (Table S3).

To corroborate the AT activity of the AT domain, we constructed a KS‐AT didomain version in which the active site serine 34 was mutated to Ala (called KS‐AT_0_). The mutated protein was then used to perform radiolabeling assays in the presence of [1‐^14^C]acetyl and [1‐^14^C]malonyl‐CoA. The labeled ACP1 products were analyzed by conformational sensitive gel electrophoresis (Fig. 4D). The results obtained indicate that the KS‐AT_0_ failed to catalyze the transfer reaction of both substrates malonyl‐CoA and acetyl‐CoA to the ACP1 domain, suggesting that the mutated serine is part of the AT active site.

Overall, these results suggest that the AT from ApPKS, like the AT from animal FAS, has the ability to load the ACP domain with both substrates, the starter (acetyl‐CoA or propionyl‐CoA) and the extender unit (most probably malonyl‐CoA).

### Substrate specificity in AT‐catalyzed transacylation reactions

**Figure 5:**
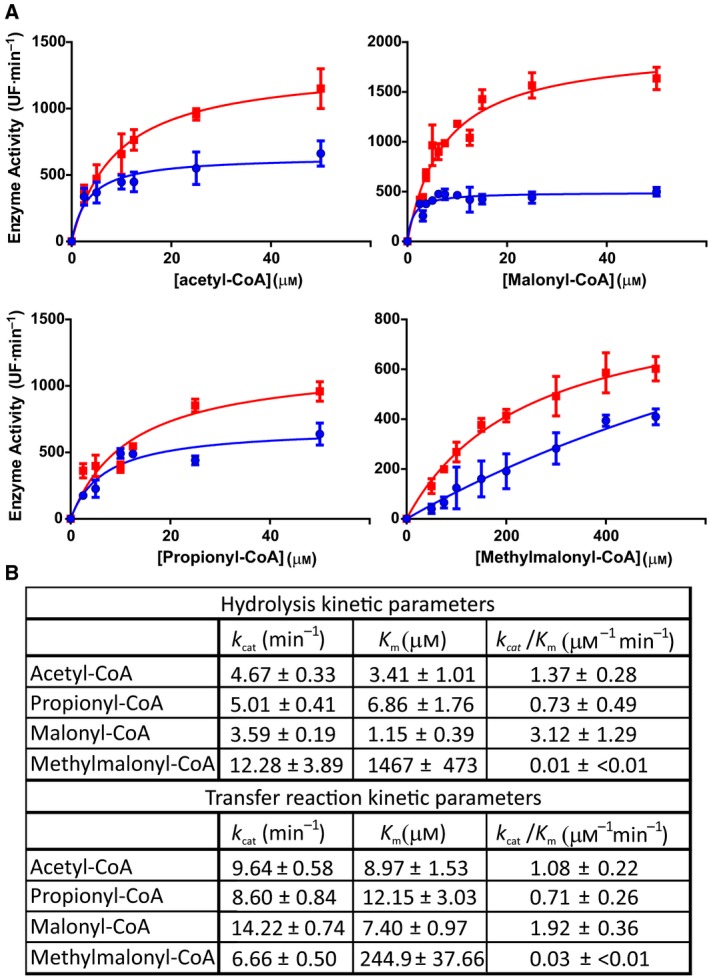
Kinetic studies of the AT‐catalyzed reactions. (A) Michaelis–Menten plots of the AT catalyzed reactions: hydrolysis (blue) and acyl transfer to ACP1 (red) of the indicated substrates. The experimental assays for hydrolytic reactions were performed under the same condition of transfer reactions but in the absence of ACP1 acceptor. Error bars reflect the standard deviation between three biological replicates. (B) The kinetic parameters listed in the table were obtained by varying the concentration of the acyl‐CoA used as substrate. All the kinetic constants listed in the table are apparent.

To further study the AT activity toward different substrates, we investigated the kinetics properties of this reaction. For this, we performed a continuous enzyme‐coupled assay usingα‐ketoglutarate dehydrogenase (αKGDH) which couples the free coenzyme A released during the transfer reaction to the reduction in the nicotinamide adenine dinucleotide (NAD^+^); the NADH formed is measured through fluorescence emission 35. Figure 5 summarizes the results obtained for hydrolytic and AT activities for the four selected substrates. The AT domain displayed high affinity and catalytic efficiency (*k*_cat_/*K*_m_) for the two starter units tested, acetyl‐CoA and propionyl‐CoA (*k*_cat_/*K*_m_ = 1.08 ± 0.22 μm ^−1^·min^−1^, and *k*_cat_/*K*_m_ = 0.71 ± 0.26 μm ^−1^·min^−1^, respectively). While for the extender units, AT showed the highest catalytic efficiency (with a *k*_cat_/*K*_m_ of = 1.92 ± 0.36 μm ^−1^·min^−1^) for malonyl‐CoA; approximately 64‐fold higher than the *k*_cat_/*K*_m_ for methylmalonyl‐CoA.

Interestingly, the data obtained for the AT hydrolytic activities for acetyl‐CoA, propionyl‐CoA, and malonyl‐CoA are in the same order of magnitude than the ones corresponding to the transacylation activities (Fig. 5). Here, the hydrolytic reaction involves the attack of the carboxyacyl−enzyme intermediate by a solvent nucleophile and not by the nucleophilic thiol from the ACP protein. Thus, a high hydrolysis rate suggests that the first half‐reaction, which involves the formation of a carboxyacyl−enzyme intermediate and release of the coenzyme A (CoASH) product, would be the rate‐limiting step that determines the substrate specificity. This data would be in agreement with a ping‐pong bi–bi mechanism which requires the acyl group binding to the AT domain 7. Furthermore, in the presence of ACP, the transfer reaction is the most important process; however, given that ACP is one of the substrates of the overall reaction and that there is a competition with the solvent as an acceptor of the acyl group, we cannot rule out the possibility that in the presence of ACP, the hydrolytic rates are lower than in its absence, being the parameters for the acyl‐AT/ACP transacylation reaction underestimated.

### Characterization of β‐ketoacyl‐ACP synthase activity of the recombinant KS domain

**Figure 6:**
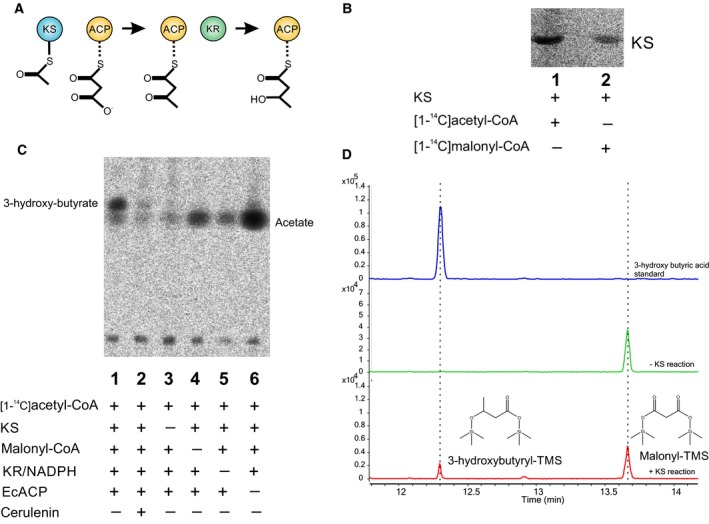
Condensation activity of KS domain. (A) Schematic representation of the condensation reaction where the acetyl group bound to the KS domain and the malonyl group bound to the ACP form the new carbon–carbon bond by Claisen condensation, given 3‐ketobutyryl‐ACP. The KR reduces this product to form 3‐hydroxybutyryl‐ACP. (B) SDS/PAGE autoradiograph of the ApPKS KS domain coincubated with radiolabeled acetyl‐CoA and malonyl‐CoA, respectively. KS can be acylated with acetyl‐CoA and to a lesser extent with malonyl‐CoA. Labeling of KS domain with malonyl‐CoA probably suggests that the KS domain may decarboxylate malonyl‐CoA or that a spountaneous decarboxylation occurs (remaining an acetyl group attached to the Cys of the active site). (C) Thin layer chromatography autoradiograph of the condensation products after alkaline hydrolysis. (D) GC‐MS analysis of the condensation products. After alkaline hydrolysis, the ACP released species which were silylated and separated by gas chromatography. Upper panel; chromatogram of a silylated 3‐hydroxybutyric acid standard. Middle panel; reaction without the KS, where no product is formed and only the substrate malonic acid is present. Lower panel; reaction where the condensation product is formed. The chemical structures of the substrate and product trimethylsilyl derivates are below the chromatographic peaks.

**Figure 7:**
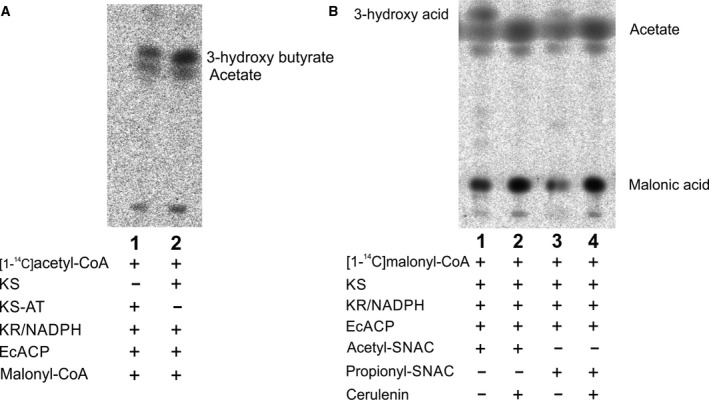
Analysis of the condensation reaction. (A) Thin layer chromatography autoradiograph after alkaline hydrolysis of reactions containing KS or KS‐AT as condensing domains. The expected product, 3‐hydroxy butyrate, and the side product, acetate, are indicated. (B) Thin layer chromatography autoradiograph after alkaline hydrolysis of reactions containing acetyl‐ and propionyl‐SNAC as starter units. The expected condensation products are 3‐hydroxy butyrate in lane 1 and 3‐hydroxy pentanoate in lane 3.

Once the functionality and substrate specificity of ApPKS AT was determined, we assayed the condensing activity of its KS domain. Guided by the substrate specificity of the AT (Figs 3 and 4), and considering the current model for the initial reaction in the fatty acid biosynthesis, in which an acetyl group attached to the active site cysteine of the KS domain is condensed with a malonyl group attached to the phosphopantheteine of the ACP domain, we started our studies by evaluating the acylation activity of the stand‐alone KS fragment using labeled [1‐^14^C]acetyl‐CoA. Figure 6B shows the labeling KS fragment; this self‐acylation of KS was already reported for other PKSs 36, 37. Once the acylation of the KS was confirmed (Fig. 6B), and self‐malonylation of the ApPKS ACP1 domain was proven to occur spontaneously (Fig. 4), we examined the condensation activity of the dissociated system by incubating the recombinant KS with unlabeled malonyl‐CoA, labeled [1‐^14^C]acetyl‐CoA, and holo‐ACP1. This reaction mix also included NADPH and the recombinant KR fragment, which should catalyze the reduction of the unstable 3‐ketobutyryl‐ACP to its corresponding stable 3‐hydroxybutyryl‐ACP. Exhaustive variations in the reaction conditions (range of incubation times from 10 min to 16 h, temperature from 15 to 37 °C, reaction volume from 10 to 100 μL, substrates concentration from 10 to 200 μm) in addition to preincubation of KS domain with acetyl‐CoA, the presence of either of the three ACP variants (ACP1, ACP2, or ACP3), the substitution of the KS domain for the KS‐AT didomain, resulted in no detection of the condensation product. Therefore, bearing in mind that the protein–protein interaction that dictates intramodule recognition in KS‐catalyzed chain elongation is not established for iterative PKS, and that the contribution of these interactions in the context of dissociated domains may differ from the one found in the full‐length protein, we decided to evaluate the condensation reaction using a type II (dissociated) ACP. In this case, we assayed the commercially pure *E. coli* ACP protein. Therefore, the subsequent condensation reaction contained KS, unlabeled malonyl‐CoA, labeled [1‐^14^C]acetyl‐CoA, *E. coli* ACP, NADPH, and KR (see Experimental procedures). The labeled compounds were detected by thin layer chromatography, after alkaline hydrolysis of the ACP‐bound products. As shown in Fig. 6C, the formation of a new product was observed when incubating all these proteins with the indicated substrates (Fig. 6C, lane 1). The reaction was inhibited by the addition of cerulenin (Fig. 6C, lane 2) and the product was not observed in the absence of any of the essential components of the reaction (Fig. 6C, lanes 3 to 6). Similar results were obtained when the condensation reaction was carried out with KS‐AT, unlabeled malonyl‐CoA, labeled [1‐^14^C]acetyl‐CoA, *E. coli* ACP, NADPH, and KR (Fig. 7A); indicating that KS domain of ApPKS is functional in either of the recombinant protein versions utilized, and suggesting that the AT and the AT postlinker are dispensable for the condensing reaction.

The identity of the condensation product, 3‐hydroxybutyryc acid, was confirmed by GC‐MS analysis of the reaction mix after silylation with N‐methyl‐N‐(trimethylsilyl)‐fluoroacetamide (MTSFA; Fig. 6D).

Finally, since the determinants of KS active site specificity toward the starter substrate remain poorly understood, we evaluated different chain length acyl‐SNACs as possible priming units. The acyl‐SNACs analyzed were: acetyl‐SNAC, propionyl‐SNAC, butyryl‐SNAC, 2‐methylbutyryl‐SNAC, and hexanoyl‐SNAC. The activity of the recombinant KS was assayed by incubating each of the acyl‐SNAC, [1‐^14^C]malonyl‐CoA, and NADPH in combination with KR and *E. coli* ACP. After reaction, alkaline hydrolysis, and acidification, TLC phosphorimaging revealed formation of a single radioactive product for the condensing reaction with acetyl‐SNAC (Fig. 7B, lane 1), and to much lesser extent with propionyl‐SNAC (Fig. 7B, lane 3). No radioactive product was detected for the longer carbon chain acyl‐SNACs (butyryl‐SNAC, 2‐methylbutyryl‐SNAC, and hexanoyl‐SNAC). These results suggest that C2 and C3 SNAC units are good KS substrates while suggesting a narrow KS substrate tolerance, at least under the assayed conditions.

## Discussion

The overall series of reactions catalyzed by PKSs and FAS systems are very similar in many aspects. For example, (a) the substrate (primer or nascent carbon chain) bound to the KS active site cysteine is condensed with the chain‐extender substrate bound to the phosphopantetheinyl arm of an ACP domain; (b) the resulting β‐ketoacyl product is then subjected to total or partial β‐carbon reduction prior to the next elongation step; and (c) all the reaction intermediates remain covalently associated with the enzyme ACP domain. Nonetheless, the organization and the implementation of these processes differ in several significant points. One of the most important difference occurs at the level of the AT domain. For example, for most iterative fungal and bacterial PKSs, it is observed that the loading of the primer and chain‐extender substrates is catalyzed by separate dedicated AT domains, for instance, several fungal PKSs have a starter unit acyltransferase domain (SAT domain) 38; thus, no competition exist between these substrates for the same AT active site. However, certain fungal HR‐PKS also have an AT domain which loads both starter and extender units. Furthermore, in bacterial modular PKSs, AT domains responsible for loading the chain‐extender substrates exhibit high specificity for the extender units, either malonyl‐ or methylmalonyl‐CoA. In contrast, in the metazoan FAS, the same AT domain is responsible for loading the starter and the extender substrates, displaying also a relaxed substrate specificity accepting precursors with 2, 3, or 4 C atoms 4, 39.

The data obtained in this study suggest that the substrate selectivity of ApPKS AT resembles that of metazoan FAS MAT domain (malonyl/acetyltransferase), being capable of catalyzing the priming and elongation transacylations. Indeed, human FAS MAT domain catalyzes the transfer of acetyl‐ and malonyl‐CoA units toward ACP with comparable kinetic parameters 40. While there was no reported MAT domain kinetic studies for methylmalonyl‐CoA as substrate until recently, it is now known that certain FAS (especially that isolated from harderian glands of mammals and the uropygial gland of waterfowls) can utilize both extender substrates *in vitro;* although malonyl‐CoA is used with two orders of magnitude more efficiently than methylmalonyl‐CoA 4. Recently, Rittner *et al*. (2018), described that murine MAT domain is polyspecific in its *in vitro* transacylation activity being capable of transferring with similar rates various acyl‐CoA‐esters including acetyl‐, malonyl‐, and also methylmalonyl‐CoA 39. As represented in Fig. 5, ApPKS AT shows higher levels of enzyme activity toward malonyl‐CoA compared with methymalonyl‐CoA (~ 64 fold). In addition, human and murine FAS MAT domains displayed about three to four orders of magnitude lower hydrolysis than transacylation rates 39, 41. Interestingly, ApPKS AT significantly differs in this aspect from metazoan FAS MAT domains and behaves more like some bacterial PKSs AT, since its hydrolytic and transacylation rates are comparable, at least in the *in vitro* conditions where it was assayed (Fig. 5). We can speculate here that hydrolysis may contribute to determine the substrate specificity as it has been suggested, for example, in DEBS AT3, where the high hydrolysis rates obtained indicated that the first step of the reaction, in which the acyl moiety is attached to the AT active site, probably is the most important bottleneck for substrate recognition 7, 39. The kinetic values obtained for ApPKS AT‐mediated reactions (Fig. 5B) are comparable with those already reported for other type I PKSs AT domains: DEBS AT3 domain for methylmalonyl‐CoA (*K*_m_ = 2 μm, *k*_cat_ = 2 min^−1^, *k*_cat_/*K*_m_ = 1 μm ^−1^·min^−1^) 42; LovB MAT domain for malonyl‐CoA (*K*_m_ = 5.4 μm, *k*_cat_ = 3.3 min^−1^, *k*_cat_/*K*_m_ = 0.62 μm ^−1^·min^−1^) 17; and the type II actinorhodin AT formalonyl‐CoA (*K*_m_ = 19 μm, *k*_cat_ = 1.6 min^−1^, *k*_cat_/*K*_m_ = 0.084 μm ^−1^·min^−1^) 43. Despite the difference in the structural organization that these enzymes exhibit, the kinetic parameters for the selection of substrates are similar, suggesting that this process could follow similar rules in such different systems.

To initiate the characterization of the ApPKS condensing activity, we develop a fully fragmented ApPKS system where the recombinant KS domain was dissected from the downstream AT domain. The results of incubation experiments with labeled substrates (Fig. 6B) confirmed an intact self‐acylation activity of this recombinant stand‐alone KS domain. Successful chain elongation in the presence of malonyl‐*E. coli* ACP further indicated that the KS domain retained condensation activity and demonstrated the feasibility of reconstituting polyketide synthase activity from disconnected domains and heterologous type II ACP. It is unclear why this reaction was not functional with none of the three different versions of the ApPKS ACPs tested. Given that we were able to measure transacylation activity between the ApPKS AT and each of these ACP domains (versions 1–3, respectively), and that the ApPKS KS and KR domains were able to form the expected product using *E. coli* ACP, we could speculate that the specific protein–protein interactions of the individual KS and ACP domains from the ApPKS could be restricting this reaction. Type II ACP proteins are flexible and suffer dynamics matching with various different partners with specific electrostatic interactions 44, 45. The overall conformation at the ACP–partner interface may be different between type I and type II ACPs. Such a difference may lay in the nature of the architecture of type I iterative enzymes which needs to maintain a comparatively rigid structure. We could also speculate on the possibility that the chaperons used here, to assist protein expression and to obtain soluble proteins, would be somehow interfering with specific KS–ACP interactions.

Regarding KS substrate specificity, metazoan FAS KS only accepts saturated acyl moieties for chain extension 46. In contrast, type I modular KS domains, such as the six KS domains in DEBS, have a wide range of substrate specificities that vary in length from diketide to decaketide; although some PKS KS domains appear to possess certain specificity with regard to different β‐carbon status 47. ApPKS KS clearly prefers short carbon‐chain acyl‐CoAs as priming unit (Figs 6 and 7B), while we did not investigate its activity toward unsaturated or partially processed longer carbon chain intermediates.

Other fascinating differences between metazoan FAS and iterative PKSs are how chain length is determined and how the tailoring domains in reducing iterative PKSs function. While in each extension cycle of metazoan FAS system the β‐ketoacyl intermediate is fully reduced, in certain reducing iterative PKSs the ER, DH, and KR have the capacity to act only on selected intermediates during the subsequent cycles of chain extension 13, 48, 49. As a consequence of this enigmatic selectivity and the unknown function specificity determinants, no product can be predicted from these megaenzymes. In this sense and particularly in the case of ApPKS, we have no clues about its product, but we can establish a comparison with MuPKS. According to Cooke *et al*. (2017) the *budgerigar* homolog MuPKS is involved in the synthesis of psittacofulvins, a yellow pigmented polyene. That study demonstrated that the heterologous expression of MuPKS led to the synthesis of a polyunsaturated fatty acid as a consequence of an inactive ER domain; thus being incapable of the final reduction of the double bound of the growing polyketide carbon chain 22. ApPKS and MuPKS proteins exhibit 81% of identity and 90% similarity. While domain prediction softwares recognize the ApPKS ER domain, given the partial conserved presence and position of the NADPH‐binding domain, the overall structure of this domain appears not to be conserved; suggesting that in our system the ER could also be inactive. Although we did not attempt to demonstrate the identity of ApPKS product, our functional and biochemical characterization and substrate specificity results support the MuPKS model. However, it is important to bear in mind that the structural rules that determine chain length and the KR, DH, and ER activities, within the overall enzyme activity, are still far from been understood in these enzymes, which hinders the prediction of the final product. Nonetheless, we could speculate, based on our data, that ApPKS would probably synthesize a polyene product similar to the product of MuPKS, but then we cannot rule out the possibility of the existence of a trans‐acting ER activity. Future studies on ApPKS, including domain deconstruction and functional heterologous expression, would provide significant advances needed for deciphering the mechanism and final product of this enzyme.

## Experimental procedures

### Media and growth conditions

*Escherichia coli* strains were grown either on solid or in liquid Luria–Bertani medium (LB; 10 g Bacto Tryptone, 5 g yeast extract, and 10 g NaCl per liter) at 37 °C and supplemented when needed with the following antibiotics: 100 μg·mL^−1^ ampicillin (Ap), 50 μg·mL^−1^ kanamycin (Km), and 20 μg·mL^−1^ chloramphenicol (Cm).

### Plasmid construction

*Escherichia coli* DH5α was used for routine cloning and subcloning 50. All the oligonucleotide primers and plasmids used in this work are listed in Table S1. ApPKS gen (Genbank ID:LOC101804178) was codon optimized and synthesized by Genescript. ApPKS was digested from plasmid pPS1 with *Nco*I and *Hin*dIII and ligated into pET28a digested with the same enzymes yielding pPS4. KS‐AT didomain was amplified using oligonucleotides KS_Fw and AT_Rv, after purification of the DNA fragment it was cloned into pGEM‐Teasy vector to yield plasmid pPS28 which was then digested with *Nhe*I and *Spe*I and the resulting plasmid was then cloned into pET28a digested with the same enzymes, the resulting plasmid was named pPS31. KS domain was subcloned from pPS4 by digestion with *Xba*I and *Mfe*I and ligation into pET28a digested with the *Xba*I and *EcoR*I, the resulting plasmid was named pPS41. KR was amplified using oligonucleotides KR_Fw and KR_Rv and the DNA fragment was then cloned into a pBluescript vector, the resulting plasmid, pPS44, was then digested with *Nde*I and *EcoR*I and ligated into pET28a digested with the same enzymes yielding plasmid pPS45. The three different ACP versions were amplified using oligonucleotides ACP1_Fw, ACP2_Fw, or ACP3_Fw and ACP_Rv, respectively. The three ACP PCR products were ligated into pGEM‐Teasy vector to yield plasmids pPS17, pPS56, and pPS57. The *Nde*I/*Eco*RI digest from these vectors were then ligated into pET2832 vector, the resulting ACP‐expression plasmids were named pPS27, pPS58, and pPS59, respectively, for ACP1, ACP2, and ACP3.

For the mutation of the active site serine of the KS‐AT didomain, overlap extension strategy was used. The corresponding two DNA fragments were generated by PCR amplification using AT_ser_ala_Fw/AT_Rv and AT_mut_Fw/AT_ala_ser_Rv oligonucleotides. The two PCR products were used as template for the following amplification using oligonucleotides AT_mut_Fw and AT_Rv. The mutated fragment was cloned into a pGEM‐Teasy vector yielding pPS34, and the serine replacement for an alanine was confirmed by sequencing. The mutated DNA fragment coding for KS‐AT_0_ didomain obtained from pPS34 was then cloned into pPS31 as *Kpn*I/*Pst*I digest.

### Protein expression and purification

For the expression of heterologous proteins, *E. coli* strains harboring the appropriate plasmids were grown at 37 °C in shake flasks in 1 L of LB medium in the presence of the corresponding antibiotics for plasmid maintenance to a A_600_ of 0.8. Plasmids containing KS, KS‐AT, and KR were transformed in *E. coli* BL21 (DE3) cells carrying plasmid pTF2. Plasmids containing ACPs were expressed in BAP1 cells 51. In all cases, expression was performed at 15 °C and 180 rpm, induced with 0.5 mm IPTG and, when needed, 10 ng·mL^−1^ tetracycline was added to induce chaperon expression. The cell pellets were resuspended in lysis buffer (50 mm Tris‐HCl, pH 7.5, 250 mm NaCl, 10% glycerol, and 1 mm PMSF) and lysed by sonication (5 × 1 min, on ice). After centrifugation at 20 000 ***g*** for 30 min, the supernatant was incubated with Ni^2+^‐nitrilotriacetic acid agarose (Qiagen, Venlo, Netherlands) for 1 h. The resin was washed with 10 column volumes of wash buffer (50 mm Tris‐ HCl, pH 7.5, 250 mm NaCl, 10% glycerol), and the bound protein was eluted with four column volumes of elution buffer (50 mm Tris‐HCl, pH 7.5, 250 mm NaCl, 250 mm imidazole). Proteins were dialyzed against 50 mm potassium phosphate buffer, 100 mm NaCl, 1 mm DTT and 10% glycerol concentrated using 3000–30 000 cutoff centrifugal filter and stored at −80 °C. ACPs were expressed as thiorredoxin fusion proteins, after purification, a 3‐h treatment with TEV protease was carried out, the cleaved His‐6x‐thiorredoxin tag and remaining fusion protein were removed by Ni^2+^ affinity. The pantetheinylation of the ACPs was confirmed by MS/MS analysis, the holo‐ACP form was about 40%.

### Phylogenetic analysis

Available selected PKS protein sequences were aligned and phylogenetic analyses were carried out by the maximum likelihood method using the program MEGA7 23, with 1000 bootstrap samplings. All the sequences were retrieved from the RefSeq database (NCBI).

### Synthesis of [1‐^14^C] Acyl‐CoAs

[1‐^14^C]Acetyl‐CoA was synthesized using baker′s yeast Acetyl‐CoA Synthetase ACS1 52 (Sigma, St. Louis, MO, USA) and [1‐^14^C]acetate (58.9 mCi·mmol^−1^, PerkinElmer, Waltham, MA, USA). The reaction mixture in a final volume of 1 mL contained 50 mm potassium phosphate buffer pH = 8, 10 mm MgCl_2_, 10 mm ATP, 1 mm DTT, 1 mm Coenzyme A, 0.5 mm [1‐^14^C]acetate and 0.3 U·mL^−1^ ACS1 (Sigma).

[1‐^14^C]Propionyl‐CoA was synthesized using the same protocol described above but starting from [1‐^14^C]propionate (56 mCi·mmol^−1^, Perkin Elmer) and using *E. coli* Propionyl‐CoA synthetase, PrpE 53.

[1‐^14^C]Malonyl‐CoA and [1‐^14^C]methylmalonyl‐CoA were synthesized starting from[1‐^14^C]acetyl‐CoA and [1‐^14^C]propionyl‐CoA, respectively; and the three subunits of the *Streptomyces coelicolor* acetyl‐CoA carboxylase complex (AccA, AccB, and AccE). This complex has activity toward acetyl‐CoA and propionyl‐CoA 54. The reaction contained 50 mm potassium phosphate buffer pH = 8, 3 mg·mL^−1^ BSA, 5 mm MgCl_2_, 5 mm ATP, 1 mm DTT, 0.5 mm [1‐^14^C]acetyl‐CoA or [1‐^14^C]propionyl‐CoA and 1 μm AccA, 1 μm AccB, and 1 μm AccE.

The four radioactive acyl‐CoAs synthetized were purified as follows: the reaction mixtures were acidified using 6 m HCl, next, the solution was loaded into a C18‐bond elute column (Agilent, Santa Clara, CA, USA). The column was washed with 10 volumes of 1 mm cold HCl and the acyl‐CoAs were eluted using a gradient of acetonitrile in 10 mm NH_4_Cl. The fractions containing the acyl‐CoA were acidified and bound to a new column. The acyl‐CoAs were eluted with 5 : 95 10 mm NH_4_Cl: ethanol, the eluted fraction was dried under a N_2_ steam, and then resuspended in 10 mm acetic acid. The final concentration was measured by absorbance at 230 nm.

### Radiolabeled transacylation assays

Labeling of enzymes were performed in 50 mm phosphate buffer pH = 7.5, 1 mm DTT, and 10% glycerol, KS‐AT was used at 2 μm, and ACP1 at 100 μm, [1‐^14^C]acyl‐CoAs were used at 50 μm. The reactions were incubated at room temperature for 10 min and quenched by the addition of SDS/PAGE loading buffer. Samples were directly loaded onto a 15% SDS/PAGE gel and electrophoresis was performed at 20 mA for 90 min. The gel was dried and analyzed using a Typhoon FLA 7000 (GE Healthcare Life Science, Chicago, IL, USA). For conformational sensitive gel electrophoresis, each transacylation assay mix was separated in 15% polyacrylamide gel containing 0.5 m urea.

### LC‐MS/MS analysis of acyl‐ACP

For the identification of the acyl‐ACP species formed in the transacylation assays, the reactions were carried out as described above but with nonradioactive acyl‐CoAs. After 10 min of carrying out reactions at room temperature, the proteins were precipitated with 10% TCA and after centrifugation the pellet was resuspended in 8 m urea. The mixture was diluted five times with 50 mm ammonium bicarbonate buffer and digested with 5 μL of 0.2 mg·mL^−1^ trypsin (Sigma) and 5 μL of 0.2 mg·mL^−1^ GluC (New England Biolabs, Ipswich, MA, USA) 16 h at 37 °C. The reaction was diluted twice in 50 mm ammonium bicarbonate buffer and 10% formic acid. Five microliters of the resulting peptide mixture was injected into a ZORBAX SB‐C18 column (50 mm × 4.6 mm × 3.5 μm Agilent) using a 1200 series Agilent HPLC. A binary gradient was formed by mixing mobile phase A (0.1% formic acid in water) and mobile phase B (0.1% formic acid in acetonitrile) at a flow rate of 0.2 mL·min^−1^. Initially mobile phase B was set to 5%, followed by a 5 min ramp to 20%, a 6 min increase to 60%, and then to 95% of mobile phase B in 2 min. The resulting ions were analyzed by an Agilent QTOF 6510 run in positive mode, pantetheinyl ejection was analyzed by tandem mass spectrometry using CID with an energy of 175 eV, the acyl‐peptide/pantetheinyl fragment transition was used to confirm the identity of the acyl‐ACP. Data analysis was performed using agilent masshunter software 6.0.

### αKGDH coupled assay

Assays were adapted from a malonyl‐CoA: ACP transacylase assay described by Molnos *et al*. 35. Specifically, assays were run in 96‐well microtiter plates (black polystyrene, flat bottom, half area, nonbinding surface, Corning, NY, USA). NADH fluorescence was monitored using a Synergy 2 Microplate Reader (BioTek, Winooski, VT, USA). Samples were illuminated with a tungsten light source and a 360‐nm filter, and fluorescence emission was monitored using a 400‐nm dichroic mirror with a 460‐nm filter. Reactions were run for 5 min using the minimum interval between measurements.

Assay components were prepared in three different solutions: solution A contained the ACP1, αKGDH, NAD^+^, TPP, and α‐ketoglutaric acid at four times their final concentration; solution B contained the acyl‐CoA substrate prepared at four times its final concentration; and solution C contained the KS‐AT didomain prepared at twice its final concentration and 0.1 mg·mL^−1^ BSA. All solutions were prepared in 50 mm sodium phosphate buffer, pH 7.6, 10% glycerol, 1 mm TCEP, and 1 mm EDTA. Solutions were added to the wells in the following order: 25 μL of solution A, 25 μL of solution B, and 50 μL of solution C, which initiated the reaction. Final assay concentrations were: 50 mm sodium phosphate, pH 7.6, 10% glycerol, 1 mm TCEP, 1 mm EDTA, 0.4 mU·μL^−1^ αKGDH, 0.4 mm NAD^+^, 0.4 mm TPP, 2 mm α‐ketoglutaric acid, and 0.05 mg·mL^−1^ BSA, acyl‐CoA concentrations were variable. The kinetic parameters of transacylation were corrected by substracting the self‐acylation of ACP1. The rate of reaction versus concentration curves were fit to the Michaelis−Menten equation using the curve‐fitting function of graphpad prism 6.0 (GraphPad Software, La Jolla, CA, USA).

### Condensation assays and thin layer chromatography

The condensation assay for thin layer chromatography (TLC) analysis contained 50 mm phosphate buffer pH = 7.5, 1 mm DTT, 10% glycerol, 4 mm NADPH, 50 μm of malonyl‐CoA/acetyl‐CoA, KS, and KR were used at 5 μm, and ACP at 100 μm, [1‐^14^C]acetyl‐CoA/malonyl‐CoA was used at 50 μm, the acyl‐CoA combinations tested are described in the main text. When indicated acetyl‐SNAC and propionyl‐SNAC were tested as possible starter units at a final concentration of 1 mm. Reactions were performed in 15 μL for 3 h at room temperature. The reaction was quenched by adding 2 μL of 3 m potassium hydroxide and heating the mixture for 20 min at 65 °C. Three microliters of 6 m hydrochloric acid was then added, the organic fraction was extracted in 200 μL of ethyl acetate and finally dried in a speedvac for 5 min. Ten microliters of ethyl acetate was added to the tube and spotted on a TLC, silica gel 60 F254 plates (0 ± 2 mm, Merck). A 60 : 2 : 6 : 10 : 22 mixture of acetone:H_2_O:chloroform:ethanol:ammonia hydroxide was used as the mobile phase, and the radiolabeled products were then visualized using a Typhoon FLA 7000 (GE Healthcare Life Science).

### Acyl‐SNACs synthesis

The N‐acetyl cysteamine (SNAC) thioesters of acetic acid, propionic acid, butyric acid, and 2‐methylbutyryc acid (SIGMA) were synthesized following the procedures reported previously by Wang *et al*. 55.

### GC‐MS analysis of condensation products

The condensation assay for GC‐MS analysis contained 50 mm phosphate buffer pH = 7.5, 1 mm DTT, 10% glycerol, 4 mm NADPH, 50 μm malonyl‐CoA, 50 μm acetyl‐CoA, KS, and KR were used at 5 μm and *E. coli* ACP at 100 μm. Reactions were performed in 50 μL for 3 h at room temperature. The samples were treated as described above. After drying in speedvac, the samples were resuspended in 50 μL of 30 mg·mL^−1^ O‐methoxyamine in anhydrous pyridine and heated at 65 °C for 30 min. Fifty microliters of N‐methyl‐N‐(trimethylsilyl)‐fluoroacetamide (MTSFA) was added and heated again at 65 °C for 30 min, the samples were then transferred to GC vial. The trimethylsilyl esters derivatives were analyzed in an Agilent G7039A gas chromatograph, equipped with a VF‐5 ms column (30 m, 0.25 mm, 0.25 μm). The oven temperature was initially held at 40 °C for 5 min and raised with a gradient of 10 °C·min^−1^ until 300 °C and held for 4 min. Helium was used as carrier gas at a flow rate of 1 mL·min^−1^. The volume of injection was 1 μL at a split rate of 1/20, the injector and detector were maintained at 225 °C. MS was carried out using a mass selective detector 5977 series operated at an ionization voltage of 70 eV.

## Authors’ contributions

MS, SC, SA, AA, and HG designed the experiments and analyzed the data. MS and SC performed all the experiments. ET helped in the design of the LC‐MS/MS experiments. AR and GL synthesize the acyl‐SNACs. MS, AA, and HG wrote the manuscript. All the authors read and approved the final version.

## Supporting information


**Table S1.** List of primers, plasmids, and strains.
**Table S2.** List of the proteins used in this work.
**Table S3.** Theoretical monoisotopic *m/z* values for the ACP species analyzed.
