# Supplementary material for: Biochemical characterization of the minimal domains of an iterative eukaryotic polyketide synthase
Source: FEBS J. 2018 Oct 25;285(23):4494–511. doi: 10.1111/febs.14675 (PMC6334511; doi:10.1111/febs.14675)
Supplement: Supplementary file 1 — Table S1. List of primers, plasmids, and strains. Table S2. List of the proteins used in this work. Table S3. Theoretical monoisotopic m/z values for the ACP species analyzed. [file FEBS-285-4494-s001.zip › febs14675-sup-0001-TableS1-S3.pdf]

FEBS PRESS
science publishing by scientists
WILEY
The FEBS Journal
www.febsjournal.org

Biochemical characterization of the minimal domains of an iterative eukaryotic polyketide synthase

Martin Sabatini, Santiago Comba, Silvia Altabe, Alejandro I. Recio-Balsells, Guillermo R. Labadie, Eriko Takano, Hugo Gramajo and Ana Arabolaza

DOI: 10.1111/febs.14675

Supporting Information

Biochemical characterization of the minimal domains of an iterative eukaryotic polyketide synthase

Martin Sabatini[1], Santiago Comba[1], Silvia Altabe[1], Alejandro I. Recio-Basells[2], Guillermo R. Labadie[2], Eriko Takano[3], Hugo Gramajo*[1], Ana Arabolaza*[1].

[1]Instituto de Biología Molecular y Celular de Rosario (IBR-CONICET), Facultad de Ciencias Bioquímicas y Farmacéuticas, Universidad Nacional de Rosario, Argentina. [2] Instituto de Química de Rosario (IQUIR-CONICET) Facultad de Ciencias Bioquímicas y Farmacéuticas, Universidad Nacional de Rosario, Argentina. [3] Manchester Centre of Fine and Specialty Chemicals (SYNBIOCHEM), Manchester Institute of Biotechnology (MIB), University of Manchester, United Kingdom.

*Corresponding author: Hugo Gramajo and Ana Arabolaza

Supplementary item	***Title format***	***Citation format***
Table	Table S1. list of primers, plasmids and strains	Table S1
Table	Table S2. list of the proteins used in this work	Table S2
Table	Table S3. *m/z* values of the ACP species analyzed	Table S3

Table S1

Primers

Primer name	Sequence	Reference
KS_Fw	ATGGCTAGCATGGAAATTGAAACGGC	This work
AT_Fw	ACATATGGGCGGTACCAACGCGCATG	This work
AT_Rv	GCTACTAGTTTCAGCTAAACACCGGG	This work
KR_Fw	ACATATGTTCGAAAACAAATGGGTG	This work
KR_Rv	TGAATTCACAGAACGTCAATACCT	This work
AT_mut_Fw	CTGGTATCAATTGCTTTGGCTTCG	This work
KS_mut_Rv	TTCCGGTTGTTTAACCTGACGA	This work
AT_ser_ala_Fw	CCGTCGGTCATgCgGTGGGCGAAA	This work
AT_ser_ala_Rw	ATTTCGCCCACcGcATGACCGACGG	This work
ACP1_Fw	ACATATGGAAGACTACATCACCTCTCTGGT	This work
ACP2_Fw	ACATATGAGCCTGAACAATCGTTTTCGC	This work
ACP3_Fw	ACATATGGGCATCCTGTTTAATCAAGATC	This work
ACP_Rv	TAAGCTTTTAGGAGCCATGTTCCGCACGAT	This work

Restriction sites are shown underlined.

Plasmids

Plasmid	Description	Reference
pET28	Vector for expression of N or C-terminal His-tagged proteins under the control of T7 promoter; Km^R	Novagen
pGEM-Teasy	Vector used for cloning 3´ A overhanging PCR products; Ap^R	Promega
pBluescript II SK (+)	Phagemid vector, Ap^R	Agilent
pTF2	Plasmid for expression of chaperons pGroEL, pGroES and Tig. Cm^R	[1,2]
pET2832	Vector for expression of N-terminal thiorredoxin fusion, cleavable by TEV protease, under the control of T7 promoter. Km^R	Diacovich L. (personal communication)
pPS1	pUC57 carrying ApPKS under the control of T7 promoter. Ap^R	This work
pPS4	pET28 carrying ApPKS under the control of T7 promoter. Km^R	This work
pPS17	pGEM-Teasy vector carrying ACP1. Ap^R	This work
pPS27	pET2832 carrying ACP1 under the control of T7 promoter. Km^R	This work
pPS28	pGEM-Teasy vector carrying KSAT didomain. Ap^R	This work
pPS31	pET28 carrying KSAT didomain under the control of T7 promoter. Km^R	This work

pPS34	pGEM-Teasy vector carrying $KSAT_0$ didomain. Ap^R	This work
pPS41	pET28 carrying KS domain under the control of T7 promoter. Km^R	This work
pPS44	pGEM-Teasy vector carrying KR domain. Ap^R	This work
pPS45	pET28 carrying KR domain under the control of T7 promoter. Km^R	This work
pPS56	pGEM-Teasy vector carrying ACP2. Ap^R	This work
pPS57	pGEM-Teasy vector carrying ACP3. Ap^R	This work
pPS58	pET2832 carrying ACP2 under the control of T7 promoter. Km^R	This work
pPS59	pET2832 carrying ACP3 under the control of T7 promoter. Km^R	This work
**Strains**		
**Strain**	**Description**	**Reference**
DH5α	*E. coli* K12 *F⁻ lacU169 (Φ80lacZΔM15) endA1 recA1 hsdR17 deoR supE44 thi-1 l2 gyrA96 relA1*	[3]
BL21 (DE3)	*E. coli F⁻ ompT gal dcm lon hsdSB (rB - mB -) λ(DE3)*	Novagen
BAP1	*E. coli F-ompT hsdSB (rB-mB-) gal dcm λ(DE3) prpRBCD:: T7prom-sfp-T7prom-prpE*	[4]

Ap^R, ampicillin resistance; Km^R, kanamycin resistance; Cm^R, cloramphenicol resistance.

Table S1. List of primers, plasmids and strains

Table S2

**Protein**	**MW (KDa)**	**6xHis Tag**	**6xHis+Thioredoxin Tag**	**Expression strain**	**protein yield (mg/L)**
KS	47.1	C-terminal	-	BL21 + pTF2	0.7
KS-AT	97.6	N-terminal	-	BL21 + pTF2	0.6
KS-AT_0	97.6	N-terminal	-	BL21 + pTF2	0.6
KR	87.8	N-terminal	-	BL21 + pTF2	1.3
6H-Trx-ACP1	22.2	-	N-terminal	BAP1	3.7
6H-Trx-ACP2	28.3	-	N-terminal	BAP1	6.5
6H-Trx-ACP3	35.6	-	N-terminal	BAP1	5.1
ACP1	7.6	-	-	BAP1	3.1
ACP2	13.6	-	-	BAP1	5.4
ACP3	20.9	-	-	BAP1	41

Table S2. List of the proteins used in this work

Table S3

**ACP species**	**Peptide**		**Pantetheinyl elimination**	
	**Chemical formula**	**$(M+3H^+)^{3+}$**	**Chemical formula**	**$(M+H^+)^+$**
Apo-ACP	$C_{106}H_{187}N_{29}O_{35}S_3$	841.7708	-	-
Holo-ACP	$C_{117}H_{209}N_{31}O_{41}PS_4$	955.1328	$C_{11}H_{20}N_2O_3S$	261.3626
Acetyl-ACP	$C_{119}H_{210}N_{31}O_{42}PS_4$	969.1363	$C_{13}H_{22}N_2O_4S$	303.3994
Propionyl-ACP	$C_{120}H_{212}N_{31}O_{42}PS_4$	973.8026	$C_{14}H_{24}N_2O_4S$	317.1534
Malonyl-ACP	$C_{120}H_{210}N_{31}O_{44}PS_4$	983.7996	$C_{14}H_{22}N_2O_6S$	347.4089
Methylmalonyl-ACP	$C_{121}H_{211}N_{31}O_{44}PS_4$	988.4714	$C_{15}H_{24}N_2O_6S$	361.4355

Table S3. Theoretical monoisotopic *m/z* values for the ACP species analyzed

References

1. Nishihara K, Kanemori M, Kitagawa M, Yanagi H & Yura T (1998) Chaperone coexpression plasmids: Differential and synergistic roles of DnaK-DnaJ-GrpE and GroEL-GroES in assisting folding of an allergen of Japanese cedar pollen, Cryj2, in Escherichia coli. *Appl. Environ. Microbiol.* **64**, 1694–1699.

2. Nishihara K, Kanemori M & Yanagi H (2000) Overexpression of Trigger Factor prevents aggregation of recombinant proteins in Escherichia coli. *Appl. Environ. Microbiol.* **66**, 884–889.

3. Hanahan D (1983) Studies on transformation of Escherichia coli with plasmids. *J. Mol. Biol.* **166**, 557–580.

4. Pfeifer B a, Admiraal SJ, Gramajo H, Cane DE & Khosla C (2001) Biosynthesis of complex polyketides in a metabolically engineered strain of E. coli. *Science* **291**, 1790–1792.
